# Unhealthy geopolitics: can the response to COVID-19 reform climate change policy?

**DOI:** 10.2471/BLT.20.269068

**Published:** 2020-11-30

**Authors:** Jennifer Cole, Klaus Dodds

**Affiliations:** a Geography Department, Royal Holloway University of London, Egham Hill, Egham, Surrey TW20 0EX, England.

## Abstract

The geopolitics of pandemics and climate change intersect. Both are complex and urgent problems that demand collective action in the light of their global and trans-boundary scope. In this article we use a geopolitical framework to examine some of the tensions and contradictions in global governance and cooperation that are revealed by the pandemic of coronavirus disease 2019 (COVID-19). We argue that the pandemic provides an early warning of the dangers inherent in weakened international cooperation. The world’s states, with their distinct national territories, are reacting individually rather than collectively to the COVID-19 pandemic. Many countries have introduced extraordinary measures that have closed, rather than opened up, international partnership and cooperation. Border closures, restrictions on social mixing, domestic purchase of public health supplies and subsidies for local industry and commerce may offer solutions at the national level but they do not address the global strategic issues. For the poorest countries of the world, pandemics join a list of other challenges that are exacerbated by pressures of scarce resources, population density and climate disruption. COVID-19’s disproportionate impact on those living with environmental stresses, such as poor air quality, should guide more holistic approaches to the geopolitical intersection of public health and climate change. By discussing unhealthy geopolitics, we highlight the urgent need for a coordinated global response to addressing challenges that cannot be approached unilaterally.

## Résumé

La géopolitique en matière de pandémie partage nombre de similitudes avec celle pratiquée face au changement climatique. Toutes deux sont liées à des problèmes complexes et urgents qui requièrent une action collective en raison de leur portée internationale, qui ne tient compte d'aucune frontière. Dans cet article, nous utilisons un cadre géopolitique pour examiner certaines tensions et contradictions observées dans la gouvernance et la coopération mondiales, et mises en lumière par la pandémie de maladie à coronavirus 2019 (COVID-19). Nous estimons que cette pandémie équivaut à un premier avertissement quant aux dangers inhérents à une faible coopération internationale. Les États de la planète et les territoires nationaux placés sous leur autorité réagissent chacun de leur côté à la pandémie de COVID-19, alors qu'ils devraient adopter une approche commune. De nombreux pays ont instauré des mesures exceptionnelles qui ont entraîné la disparition des partenariats existants au lieu de les renforcer. Fermer les frontières, restreindre la mixité sociale, acheter des fournitures médicales pour les hôpitaux à l'intérieur du pays et subventionner l'industrie et les commerces locaux peuvent certes apporter des solutions à l'échelle nationale, mais ces décisions ne s'attaquent pas aux problèmes stratégiques à l'échelle mondiale. Pour les pays les plus pauvres de la planète, la pandémie s'ajoute à une longue liste de défis exacerbés par la pression qu'exercent la pénurie de ressources, la densité de population et le dérèglement climatique. L'impact disproportionné de la COVID-19 sur celles et ceux qui subissent déjà un stress environnemental, dû par exemple à la mauvaise qualité de l'air, devrait servir de guide à une série d'approches holistiques reflétant le rapport entre santé publique et changement climatique. En abordant le thème d'une géopolitique néfaste, nous attirons l'attention sur l'urgence d'une réponse globale coordonnée qui permettra de relever des défis qui ne peuvent être affrontés unilatéralement.

## Resumen

La geopolítica en materia de pandemias comparte muchas similitudes con la del cambio climático. Ambos son problemas complejos y urgentes que exigen una acción colectiva debido a su alcance global y transfronterizo. En este artículo utilizamos un marco geopolítico para examinar algunas de las tensiones y contradicciones en la gobernanza y la cooperación mundiales que pone de manifiesto la pandemia de la enfermedad del coronavirus de 2019 (COVID-19). Consideramos que la pandemia proporciona una alerta temprana de los peligros inherentes a una cooperación internacional debilitada. Los Estados del mundo, con sus distintos territorios nacionales, están reaccionando individualmente en lugar de colectivamente a la pandemia de la COVID-19. Muchos países han introducido medidas extraordinarias que han cerrado, en lugar de abrir, la asociación y la cooperación internacionales. El cierre de fronteras, las restricciones a la mezcla social, la compra interna de suministros de salud pública y las subvenciones a la industria y el comercio locales pueden ofrecer soluciones a nivel nacional, pero no abordan las cuestiones estratégicas mundiales. En el caso de los países más pobres del mundo, las pandemias se suman a una lista de otros problemas que se ven agravados por las presiones de la escasez de recursos, la densidad demográfica y los trastornos climáticos. El impacto desproporcionado de la COVID-19 en quienes viven con tensiones ambientales, como la mala calidad del aire, debería orientar los enfoques más holísticos de la intersección geopolítica de la salud pública y el cambio climático. Al examinar la geopolítica dañina, ponemos de relieve la urgente necesidad de una respuesta mundial coordinada para hacer frente a los desafíos que no pueden abordarse unilateralmente.

## ملخص

تتداخل الجغرافيا السياسية للأوبئة مع التغير في المناخ. وكل منهما مشكلتان معقدتان وعاجلتان تتطلبان العمل الجماعي في ضوء نطاقهما العالمي والعابر للحدود. نستخدم في هذا المقال إطار عمل جغرافي سياسي لفحص بعض التوترات والتناقضات في الحوكمة والتعاون العالميين، والتي كشف عنها مرض فيروس كورونا 2019 (كوفيد 19). نحن نزعم أن الوباء يمثل إنذارًا مبكرًا عن الأخطار الكامنة في التعاون الدولي الواهن. تتفاعل دول العالم، بأقاليمها الوطنية المتميزة، بشكل فردي أكثر منه جماعي مع وباء كوفيد 19. لقد قدمت العديد من الدول إجراءات استثنائية أدت إلى إغلاق الشراكة والتعاون الدوليين بدلاً من الانفتاح عليهما. إن كل من حالات إغلاق الحدود، والقيود المفروضة على الاختلاط الاجتماعي، والمشتريات المحلية لمستلزمات الصحة العامة، والإعانات للصناعة والتجارة المحلية، قد تقدم حلولاً على المستوى الوطني لكنها لا تعالج القضايا الاستراتيجية العالمية. وبالنسبة لأكثر دول العالم فقراً، فإن الأوبئة تشارك في قائمة من التحديات الأخرى التي تفاقمت بسبب ضغوط الموارد الشحيحة، والكثافة السكانية، واضطراب المناخ. إن التأثير غير المتناسب لكوفيد 19 على أولئك الذين يتعايشون مع الضغوط البيئية، مثل نوعية الهواء الرديئة، يجب أن يوجه أساليب أكثر شمولية للتداخل الجغرافي السياسي بين الصحة العامة وتغير المناخ. نقوم من خلال مناقشة الجغرافيا السياسية غير الصحية، بتسليط الضوء على الحاجة الملحة لاستجابة عالمية منسقة لمواجهة التحديات التي لا يمكن التعامل معها من جانب طرف واحد.

## 摘要

流行病的地理政治现象与气候变化之间存在交汇点。由于全球性和跨国界的特点，两者都属于迫切需要采取集体行动来解决的复杂问题。在本文中，我们采用地理政治框架来研究新型冠状病毒肺炎（新冠肺炎）大流行所揭示的全球性治理与合作方面的紧张局势和矛盾冲突。我们认为，对于国际合作削弱所蕴藏的危机，本次大流行发挥了预警作用。世界各国各自拥有其国家领土，在应对新冠肺炎大流行这个问题上，当前各国各自独立地采取应对措施，而没有团结一致。许多国家采取了离奇的措施，这些措施不仅没有推进国际伙伴关系与紧密合作，反而导致合作破裂与终止。关闭边境、限制社会阶层混居、在国内购买公共卫生用品以及对当地工商业提供补助可能在国家层面提供了解决方案，但无法解决全球性战略问题。对世界上最贫穷的国家而言，流行病还伴随着一系列其他问题的出现，而资源短缺、人口密度和气候破坏方面的压力导致情况进一步恶化。新冠肺炎对承受环境压力（例如空气质量差）地区的影响不均衡，应该针对公共卫生与气候变化的地理政治交汇点开展更多整体研究。通过讨论不健康的地理政治现象，我们强调，目前迫切需要采取协调一致的全球性应对策略来解决无法单方面克服的挑战。

## Резюме

Курсы геополитики в области пандемии и изменений климата пересекаются. Обе проблемы являются сложными и требуют неотложного внимания и коллективных действий в свете их глобального и трансграничного масштаба. В данной статье авторы используют геополитическую структуру для изучения напряженности и противоречий в глобальном управлении и сотрудничестве, которые были выявлены во время пандемии коронавирусного заболевания 2019 г. (COVID-19). Авторы утверждают, что пандемия является ранним предупреждением об опасностях, связанных с ослаблением международного сотрудничества. Мировые государства с раздельными национальными территориями реагируют на пандемию COVID-19 индивидуально, а не коллективно. Множество стран ввели чрезвычайные меры, которые скорее закрыли, а не открыли возможности для международного партнерства и сотрудничества. Закрытие границ, ограничения на социальное смешивание, закупки товаров общественного здравоохранения на внутреннем рынке и субсидии для местной промышленности и торговли могут предлагать решения на национальном уровне, но они не решают глобальных стратегических проблем. В беднейших странах мира пандемия пополнила список других проблем, которые усугубляются нехваткой ресурсов, плотностью населения и дестабилизацией климата. Непропорциональное воздействие COVID-19 на людей, живущих в условиях экологических стрессов, таких как плохое качество воздуха, должно привести к более целостным подходам к геополитическому пересечению проблем общественного здравоохранения и изменения климата. Обсуждая нездоровую геополитическую ситуацию, авторы подчеркивают настоятельную необходимость в скоординированных глобальных ответных мерах для решения проблем, которые невозможно решить в одностороннем порядке.

## Introduction

The pandemic of coronavirus disease 2019 (COVID-19) is a global public policy challenge. While there is little genetic difference in the strains of severe acute respiratory syndrome coronavirus 2 (SARS-CoV-2) virus currently circulating,^1^ the impact of the pandemic has varied considerably from country to country, exposing inequalities and vulnerabilities in health care, political systems and economies at local, national and international levels. Disparities in infection fatality rates and nations’ varying ability to manage the outbreak^2^ point to socioeconomic and geopolitical factors that have as much impact on the progress of the COVID-19 pandemic as do the characteristics of the SARS-CoV-2 virus.

The pandemic exposes the reality of world politics – that no world government exists to impose consistent, proportionate and uniform public health measures. The world’s states, with their distinct national territories, are reacting individually rather than collectively to this pandemic. Border closures and travel bans create tensions as countries label others, but resent being labelled themselves, as unsafe or risky places to travel.^3^

As the discussions about (re)emerging diseases have recognized since the late 1980s and 1990s,^4^^,^^5^ open borders and enhanced mobility of populations carry global risks and vulnerabilities, from uncontrolled migration to rapid disease diffusion. Compounding matters further, the rapid pace of climate change makes it even more likely that the global community will face yet more transnational challenges, including large-scale forced population movements, over the coming decades.^6^

The COVID-19 pandemic is an example of what has been called a super-wicked problem.^7^ These are problems characterized by the urgent need to find a solution; where the solution to the problem rests with those who are causing it; where central authority to address the problem is weak; and where actions taken today can store up problems for the future. Pandemics share these similarities with climate change; both issues are complex and urgent, and if left unchecked will continue to place huge burdens on the future health and well-being of humanity and the biosphere. While neither problem respects national borders, the impact of pandemics and climate change within countries is highly dependent on the intersection of laws, policies and social factors.^2^^,^^8^ Collective human action is needed to resolve these super-wicked problems. Past experience of problem-solving at the international level, however, is a reminder that some new approaches are needed. For example, countries are struggling to meet the targets of the United Nations sustainable development goals and the Framework Convention on Climate Change.

We use a geopolitical framework to examine some of the tensions and contradictions in global governance and cooperation that are revealed by the COVID-19 pandemic. We propose guidance for policy-makers and public health professionals that will help address future challenges from climate change, as well as infectious diseases.

## Geopolitical tensions

The COVID-19 outbreak has exacerbated political tensions within and beyond state borders,^9^ notably between China and the United States of America (USA). The existing literature on the geopolitical determinants of health has focused on the inadequacy of health systems in low- and lower-middle income countries, particularly those still recovering from years of internal conflict.^10^ There is also discussion on the population-level health risks in countries with little choice but to accept economic opportunities that pose a high risk of pollution.^11^ More work is needed, however, on how public health challenges can affect public opinion. The politics of nationalism and of populist debates that undermine confidence in expert opinion are reminders that just because there appear to be powerful incentives to cooperate on public health challenges, it does not mean that cooperation will automatically follow.^12^^,^^13^ Narratives of climate change are already highly politicized, such as by categorizing certain countries as net emitters of carbon dioxide or assigning historic burdens of responsibility for environmental impact to countries. It has been noted that world leaders who have responded more slowly and less effectively to the current pandemic, resulting in high case numbers and deaths within their borders, also tend to play down their country’s responsibility for preventing climate change.^14^

An increase in the incidence of vector-borne and zoonotic disease emergence and transmission is a long-predicted consequence of environmental change.^15^ Like other climate-induced disasters that threaten the well-being of national populations, diseases have no respect for national borders. This fact exposes the myth of national sovereignty:^16^ the notion that any government enjoys exclusive control over its national territories. Yet a notable element in many countries’ reactions to the COVID-19 pandemic has been the introduction of extraordinary measures that have closed down, rather than opened up, international partnership and cooperation. Border closures, restrictions on social mixing, domestic purchase of public health supplies and subsidies for local industry and commerce may offer some protection from local infection spread at the national level but they do not address the global strategic issues. The pandemic is leaving virtually every country in the world with significant burdens of economic debt that are likely to disadvantage long-term plans for climate change mitigation, jeopardize sustainable transport plans and complicate energy transition. The consumption of plastic, for example, has risen during the pandemic because of demand for plastic face shields, facemasks and sanitary packaging.^17^

Since the 1990s, open borders have largely been championed by governments and businesses eager to enjoy a global marketplace. This optimistic vision of unrestricted population movements has been shattered by the COVID-19 pandemic. Responses that are overly focused on security in response to adverse events – for example after terrorist attacks – are now being applied to pandemics, migration and climate change. Over the last two decades, governments around the world have already invested more in security barriers and border walls. The pandemic is likely to further reinforce this trend. Public health is rarely approached in isolation: those who are already concerned about cross-border migration of people for asylum or work may view migrants as imperilling the nation-state by importing disease.^18^ Even the most privileged people cannot escape the impact of COVID-19 entirely.^19^ Yet those existing at the edges of society – such as illegal migrants whose health is already affected disproportionately by poor access to affordable housing and public services – are less likely to be able to access medical assistance.^20^

COVID-19’s disproportionate impact on those living with environmental stresses such as poor air quality^21^ should guide more holistic approaches to the geopolitical intersection of public health and climate change.

We are living in what has been called the Anthropocene.^22^ This era is defined as a period in the Earth’s history in which – unlike previous geological time periods – human beings are having more impact on the environment than are biological and geophysical processes. Climate change in the Anthropocene era threatens to cause nonlinear and abrupt changes in environmental and atmospheric conditions.^23^^,^^24^ These changes will place further pressures on our current system of international cooperation which has been put to the test by the COVID-19 pandemic.

## Understanding public health geopolitics

A geopolitical framework provides opportunities to better understand three interconnected thematic areas. The first theme is the working geopolitics of institutions. The early years of the 21st century have been described as a post-liberal international order,^25^ marked by the return of political rivalries between China, the Russian Federation and the United States, which creates challenges to international coordination and cooperation. War-like language and blame narratives around the origin and spread of the virus make it more difficult for an expert body such as the World Health Organization (WHO) to demonstrate international leadership, to mobilize expert advice and to make recommendations.

The early history of the COVID-19 pandemic shows how the geographical framing of an existential threat and its apparent origins can become geopoliticized. From the first announcement of a novel virus by WHO on 4 January 2020 to 11 March 2020, when WHO announced the COVID-19 outbreak as a pandemic, the geopolitical framing mattered. Such framing occurred despite the formal declaration of a public health emergency of international concern on 30 January 2020. Politicians and the mass media who labelled SARS-Cov-2 as the China virus or China flu, against the explicit desire of WHO,^26^ actively encouraged conspiracy theories, including allegations of secretive and dangerous research at laboratories in Wuhan, the centre of the outbreak of COVID-19. Such geographical framings that encourage anti-Chinese sentiment are likely to imperil information-sharing and collective action and must be resisted.

Second, infectious disease can only be overcome globally if it is tackled in every country.^27^ The environmental economist Elinor Ostrom, in her seminal work *Governing the commons*,^28^ suggested that long-term sustainability needs institutional frameworks to better understand the complex relationship between users and resource systems. For both climate change and global health, therefore, we need to recognize that the pursuit of a narrowly national or strategic advantage generates real dangers. Ostrom was clear that these super-wicked problems demand expert knowledge of how social, economic and ecological systems interact, and about collective choice rules and shared moral and ethical standards. Neither pandemics nor the adverse effects of climate change can be kept at national borders.

The return of competition among geopolitical power blocks has paradoxical consequences. On the one hand, power imbalances can encourage health diplomacy in the form of medical and humanitarian assistance or intervention to areas of respective interest (such as the Russian Federation providing medical supplies to Serbia, or China sending supplies to Italy). On the other hand, competition can accelerate concerns about supply-chain dependencies, the protection of vaccine supply by nationalist governments for their own citizens, and demands for greater national self-sufficiency. The COVID-19 pandemic has placed more pressure on national governments to protect their national economies and supply chains. By prioritizing strategies to address the immediate concerns of public health, climate change mitigation may be harder in the future.

The pandemic has, however, provided an interesting confluence of public–private health diplomacy and displays of soft power. Non-state actors such as nongovernmental organizations, philanthropists and private companies have emerged to fill gaps where federal or national governments have failed to provide for their own citizens. The Chinese government has mobilized its resources to send gifts of COVID-19 resources, including respirators and surgical face masks, around the world, while individual philanthropists have been active throughout the pandemic in donating SARS-CoV-2 testing kits to Africa, Italy and the USA.^29^ Pleas have been made to world leaders to be mindful of their “natural desire to put their own people first”^30^ and to come to early agreements not only with each other but with the private sector, so that money spent on tackling the pandemic can be spent most efficiently. The role of such actors in the global commons of climate change and public health warrants further consideration.

Third, another sort of geopolitics is emerging: one that is shaped by rebellion and resistance. In terms of global health and climate change, there are a growing number of international nongovernmental organizations that call for greater global cooperation in tackling not only ill health but the economic and environmental drivers that lie beneath it. Such organizations include the Planetary Health Alliance,^9^ the EcoHealth Alliance^31^ and the International Union for the Conservation of Nature.^32^ Memberships of these organizations often cross traditional geopolitical borders, as do those of disruptive environmentalist movements such as Extinction Rebellion. What these organizations share is a desire to cut across national borders and encourage more global and cosmopolitan sensibilities, driving ground-up responses that can meet and merge with more strategic approaches. In effect, people have argued that these movements achieve what Ostrom called for: a moral consensus over the best course of action and practical rules that everyone is willing to follow.^33^

## Geopolitics of public health leadership

WHO continues to endure global scrutiny during the COVID-19 pandemic.^2^ The organization has previously been accused of over-reacting to the H1N1 influenza virus pandemic in 2009^34^ and of being too slow in responding to the Ebola virus disease outbreak in Africa in 2014.^35^ WHO has a challenging diplomatic role. The power of WHO lies in its advisory and convening abilities. WHO is a single point of contact for the world’s health challenges that should be able to leverage a collective response from its entire membership and to direct its resources to particular Member States who need help. The USA’s senior public health adviser has called for WHO to be strengthened as a way to improve collective action rules and coordination.^36^

This relationship between geopolitics and the global management of public health also offers insights for leadership in climate change. The ability to negotiate state borders, overcome defensive nationalism and counter anti-science narratives^37^ will be integral to managing both public health crises and climate change. WHO has been criticized for its handling of the early phase of the COVID-19 pandemic,^38^ yet it has no power to impose or enforce actions on its Member States. Most of WHO’s funding is composed of voluntary contributions and pledges.^39^ WHO can only act as a conduit of information, which countries may or may not choose to heed. This information can and does become entangled in the complexities of prevailing geopolitical relationships and contexts. How much nations trust each other – and trust the nation that first highlights an emerging challenge – can influence their behaviour. International agencies such as WHO or the United Nations Framework Convention on Climate Change can also become compromised. WHO’s attempts to eradicate polio in Pakistan are still hampered by suspicion and conspiracy theories within the local communities.^40^ Climate change programmes must guard against becoming involved in partisan geopolitical actions.

Collective action against global threats is possible and public support for such action can be easier to secure when the threats are more immediate and where results are likely to be identifiable in a shorter time frame. There are lessons to be learnt from the recent history of how environmental protection and human health are connected. The most successful environmental treaty so far has been the Montreal Protocol.^41^^,^^42^ Like SARS-Cov-2, a single issue (release of chlorofluorocarbons gases into the atmosphere) presented a threat to health from which high-income and more powerful countries had no better defence than the poorest countries. Action was needed immediately to avoid risks to health. Other positive examples of global cooperation on health include the eradication of smallpox^43^ and the fast-tracked development of drugs for acquired immunodeficiency syndrome.^44^ An example of global cooperation on health and environment was the recognition that laws outlawing dichlorodiphenyltrichloroethane pesticides in international treaties needed to make exceptions for regions of the world where the burden from malaria was too high.^45^

Threats to global public health provide a clear reason to act, whether the threat itself comes from pathogenic viruses and bacteria or from environmental damage. The danger, however, is that the role of the environment in health may be forgotten as the focus falls on pharmaceuticals and other medical fixes rather than addressing the root causes of (planetary) ill health. However, we must not forget that susceptibility to COVID-19 has been exacerbated by air pollution,^21^^,^^46^ and by obesity and diabetes^47^^,^^48^ linked to food and social systems that challenge the ability of the poor and disadvantaged to live healthily.

Pandemics offer multiple lessons on how to maximize opportunities for local and regional leadership, including scope for regional expertise on disease control and public health planning (such as the African Centre for Disease Control in Addis Ababa, Ethiopia, set up in the wake of the 2013–2016 Ebola virus disease outbreak) and for overcoming the challenges of coordinating and funding for prevention and planning.

## Geopolitics, health and climate change

The effects of the COVID-19 pandemic will be with us for many years to come. It is premature to speak of what will emerge when the pandemic recedes. There will be enduring health inequalities wherever public health infrastructures are underfunded and poorly equipped. For the poorest countries of the world, pandemics join a list of other challenges that are exacerbated by pressures of scarce resources, population density and climate disruption. It is perhaps this last factor with which the geopolitics of COVID-19 will most clearly intersect. Climate change and its drivers – particularly biodiversity loss and land-use change – makes the emergence of novel infectious diseases and their spread more likely to happen in poorer, rural communities,^49^ often far from centres of power. The COVID-19 pandemic is exposing systemic weakness within states and highlighting the limits on collective action.^28^

Timely action matters, since vulnerabilities inherent in Anthropocene geopolitics need to be addressed immediately, before environmental damage is irreversible.^9^ There is an imperative to act now but also to anticipate how this might influence and impact on the future. Attempts to impose further partition, bordering and appropriation delay the challenges of controlling the pandemic but do not overcome them. As a pre-emptive action on the transmission of COVID-19, shutting borders to movement of people might bring short-term relief but it will not make the planet as a whole healthier. Spatial control will never completely protect the wealthiest people from transnational threats such as air pollution or infectious diseases. While shutting down borders and imposing quarantine can be highly effective for containing disease spread, effective prevention of future pandemics demands long-term societal transformation that emphasizes the same adaptation, mitigation and resilience needed to address climate change. A vaccine for COVID-19 will not be a quick and easy solution to the challenges of degraded environments that create and exacerbate ill health. Unless climate change is tackled, far more permanent damage will be inflicted on the environment and on societies, including by future pandemics. There will be no respite unless we act now.

The current pandemic is thus giving us lessons on how geopolitical challenges might play out in future and where pressures are already accumulating. Countries have moved quickly to protect their own citizens, such as by seeking to outbid rivals for personal protective equipment or to gain exclusive access to vaccines for their own population.^50^ Public health emergencies have been used to lock down borders and further imperil the rights of refugees and asylum seekers.^51^ We might be entering into a new era of extraordinary measures where there is little to no incentive to return to the previous norms of international cooperation. Virtual meetings of diplomats and negotiators online leave less space for the in-person social and intimate interactions that help to construct shared understandings and practices.

The pandemic exposes the gaps in collective governance that are already apparent in climate change diplomacy; it places further pressure on the cooperation and collaboration the world strived for following the Second World War, when institutions such as the United Nations and WHO took on their current structures.^25^ The challenge for geopolitics, and where it can inform public health, is how to adjust to a world which is undergoing fundamental change in its political alliances as well as its ecological environments. States such as Canada and the Russian Federation worry about their strategic presence in an Arctic region affected by abrupt change. In response to other environmental pollutants, treaties such as the Rotterdam Convention on Prior Informed Consent, Stockholm Convention on Persistent Organic Pollutants, and the Basel Convention on the Control of Transboundary Movements of Hazardous Wastes and their Disposal^52^ sought to limit damaging actions and empower vulnerable communities and countries. Future pandemic preparedness may require the same kind of action.

## Conclusion

People cannot socially distance from the environmental damage that has been such a feature of the Anthropocene era and which has been implicated in the emergence and spread of COVID-19.^53^^,^^54^ More openness and sharing about threats emerging within countries’ own borders are needed to provide early warnings to the world; more collaboration and resource sharing to tackle it; and more protection of the natural environments on whose health our own health so strongly depends.

The geopolitics of public health and climate change intersect. We believe that a geopolitical framework is essential to understanding the capacity and willingness of states and the public to engage with super-wicked problems. The longer-term impacts of climate change risk amplifying the short-term impacts of pandemics as governments around the world seek to rebound after the costly interventions COVID-19 has required. We need to be aware that collective action, even when it appears obvious to many, cannot be taken for granted.
